# C4/5 Facetectomy Is Associated With Improved Recovery of C5 Palsy in Severe Foraminal Stenosis After Posterior Cervical Decompression and Fusion

**DOI:** 10.7759/cureus.109458

**Published:** 2026-05-22

**Authors:** Yusuke Nakamura, Sohei Murata, Taisuke Yabe, Hiromu Ito

**Affiliations:** 1 Department of Orthopedic Surgery, Kurashiki Central Hospital, Okayama, JPN

**Keywords:** c5 palsy, degenerative cervical myelopathy, facetectomy, foraminal stenosis, posterior decompression and fusion

## Abstract

Introduction: C5 palsy is a disabling complication after posterior cervical decompression and fusion (PCDF). Whether C4/5 facetectomy reduces the incidence or improves the outcomes of C5 palsy remains uncertain. Therefore, this study aimed to (1) test the association of C4/5 facetectomy with the incidence and recovery of C5 palsy and (2) identify radiological risk factors for C5 palsy.

Methods: A single-center retrospective cohort study was conducted between April 2013 and January 2024. Adults with degenerative cervical myelopathy, including ossification of the posterior longitudinal ligament, who underwent PCDF that included C4/5 were included. Deltoid manual muscle testing (MMT) was assessed preoperatively at discharge and at the final follow-up. Axial CT was used to measure the bilateral C4/5 foraminal diameter (FD). MRI was used to evaluate posterior cord shift at C4/5 and intramedullary T2 high-signal intensity (T2HI) at C3/4 and C4/5. C5 palsy was defined as a decrease from MMT grade ≥3 to ≤2; recovery was defined as improvement to MMT grade ≥3 at the final follow-up. A prespecified severe stenosis subgroup was defined as FD<2.6 mm. Group comparisons included Fisher’s exact test and unpaired t tests; multivariable logistic regression was used to identify risk factors.

Results: Ninety-four patients were included; C5 palsy occurred in 20 (21.3%) patients. Facetectomy was not associated with C5 palsy incidence. T2HI at C3/4 was independently associated with C5 palsy (OR: 3.11, 95% CI: 1.05-9.74; p=0.04). In the severe stenosis subgroup, facetectomy did not reduce the incidence of C5 palsy but was associated with improved recovery from C5 palsy (100% (7/7) vs. 25% (1/4); p=0.02).

Conclusions: In patients who underwent PCDF including C4/5, C4/5 facetectomy was not associated with a lower occurrence of postoperative C5 palsy. However, in patients with severe preoperative foraminal stenosis, facetectomy was associated with improved recovery after C5 palsy occurred. T2HI at C3/4 was independently associated with C5 palsy development. These findings do not support routine facetectomy for prophylaxis. The observed association between facetectomy and improved recovery in selected patients with marked foraminal stenosis should be interpreted as exploratory, given the retrospective design and small subgroup size.

## Introduction

C5 palsy is a well-recognized postoperative complication of cervical spine surgery that can cause meaningful -- although often transient -- impairment in activities of daily living; its overall incidence is approximately 5% across approaches, with higher odds after posterior decompression than after anterior procedures in patients with degenerative cervical myelopathy (DCM) [1-3]. A systematic review comparing posterior techniques has suggested that laminoplasty, compared with posterior cervical decompression and fusion (PCDF), is associated with a lower incidence of C5 palsy [2,4]. In some posterior fusion series, rates have been reported to be as high as 4%-30%, underscoring the need for prevention strategies [5-10].

Proposed risk factors include older age, preoperative myeloradiculopathy, and intramedullary T2 high-signal intensity (T2HI) on MRI, with a narrowed C4/5 foramen (foraminal stenosis) receiving particular attention [1,2,5,8-11]. Prophylactic C4/5 foraminotomy has been advocated by several groups and may reduce the incidence of C5 palsy in selected populations, although the results remain mixed [12-16]. Nevertheless, C5 palsy occurs despite foraminotomy, especially when stenosis is severe or morphologically unfavorable, suggesting that limited posterior foraminotomy can be insufficient in some cases [13,15]. In support of this concept, Gu et al. reported that patients with parallel-shaped cervical foraminal stenosis had worse outcomes after posterior cervical foraminotomy than those with V-shaped stenosis, probably because adequate decompression was more difficult to achieve in parallel-shaped foramina [13].

Compared with standard foraminotomy, C4/5 facetectomy may provide wider posterior decompression of the C5 nerve root at the C4/5 foramen. In this study, C4/5 facetectomy was defined as complete resection of the C4/5 facet joint, extending laterally to the lateral margin of the facet joint and craniocaudally from the C4 pedicle to the C5 pedicle. However, whether this wider decompression prevents postoperative C5 palsy or improves recovery following palsy onset in patients undergoing PCDF has not been systematically evaluated. We therefore investigated whether C4/5 facetectomy was associated with the incidence of C5 palsy and recovery following C5 palsy onset in patients with DCM undergoing PCDF. We also evaluated radiological risk factors and performed a prespecified exploratory subgroup analysis in patients with severe C4/5 foraminal stenosis.

## Materials and methods

Study design and patient population

This retrospective study was approved by the Institutional Review Board of Kurashiki Central Hospital (Approval No. 4498). The requirement for written informed consent was waived because of the retrospective nature of the study, and an opt-out method was used. We conducted a single-center retrospective cohort study of patients who underwent PCDF, including the C4/5 segment, between April 2013 and January 2024. Eligible patients had degenerative cervical disease, including cervical spondylotic myelopathy (CSM) and ossification of the posterior longitudinal ligament (OPLL), and underwent PCDF in which C4/5 was included among the fused levels. Patients who underwent surgery for tumors, trauma, or infection were excluded.

Surgical procedure

Surgery was performed for patients with DCM, with or without radiculopathy. The decision to add posterior fusion was based on factors such as cervical kyphotic alignment and K-line status in patients with OPLL. When kyphotic correction was required, correction was limited to in situ fixation achieved by surgical positioning. No anterior or posterior release procedures were performed in this cohort. All procedures were performed using a posterior-only approach with screw-rod fixation.

**Figure 1:**
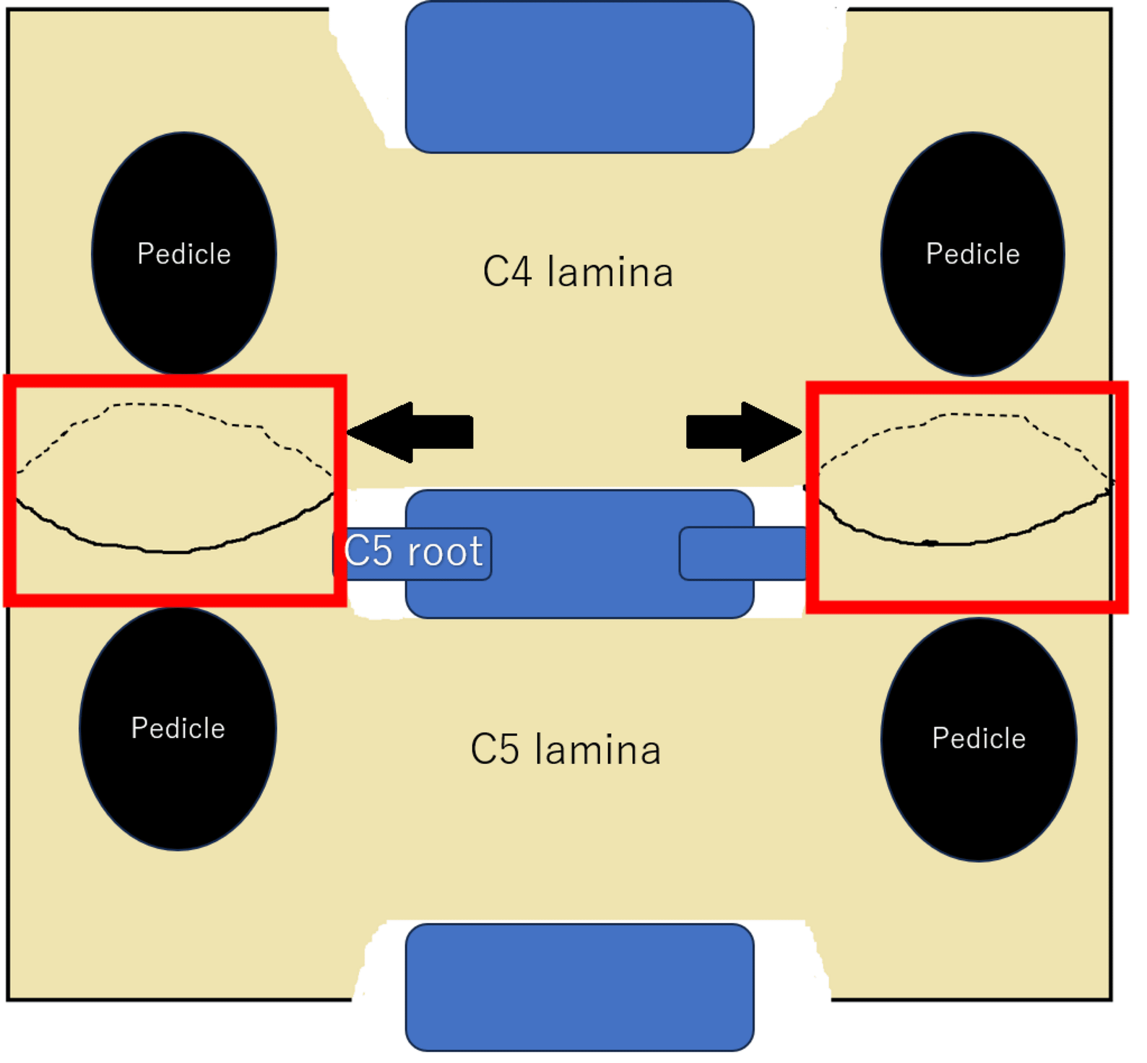
Schematic illustration of C4/5 facetectomy Black circles indicate the pedicles, and blue structures indicate the spinal cord and exiting C5 nerve roots. The red frame indicates the extent of C4/5 facetectomy. C4/5 facetectomy was defined as complete resection of the C4/5 facet joint, extending laterally to the lateral margin of the facet joint and craniocaudally from the C4 pedicle to the C5 pedicle.

C4/5 facetectomy was defined as the complete resection of the C4/5 facet joint. The lateral extent of resection reached the lateral margin of the C4/5 facet joint, and the craniocaudal extent of decompression extended from the C4 pedicle to the C5 pedicle (Figure 1). The indication for C4/5 facetectomy was not standardized during the study period. In general, C4/5 facetectomy was considered based on preoperative C5 radicular symptoms, radiographic C4/5 foraminal stenosis, and the surgeon’s intraoperative judgment.

Clinical data collection

The following clinical data were collected from medical records: age, sex, follow-up period, number of fused levels, and whether facetectomy at C4/5 was performed. Deltoid muscle strength was evaluated using the manual muscle test (MMT) preoperatively, at discharge, and at the final follow-up.

Radiological assessment

**Figure 2:**
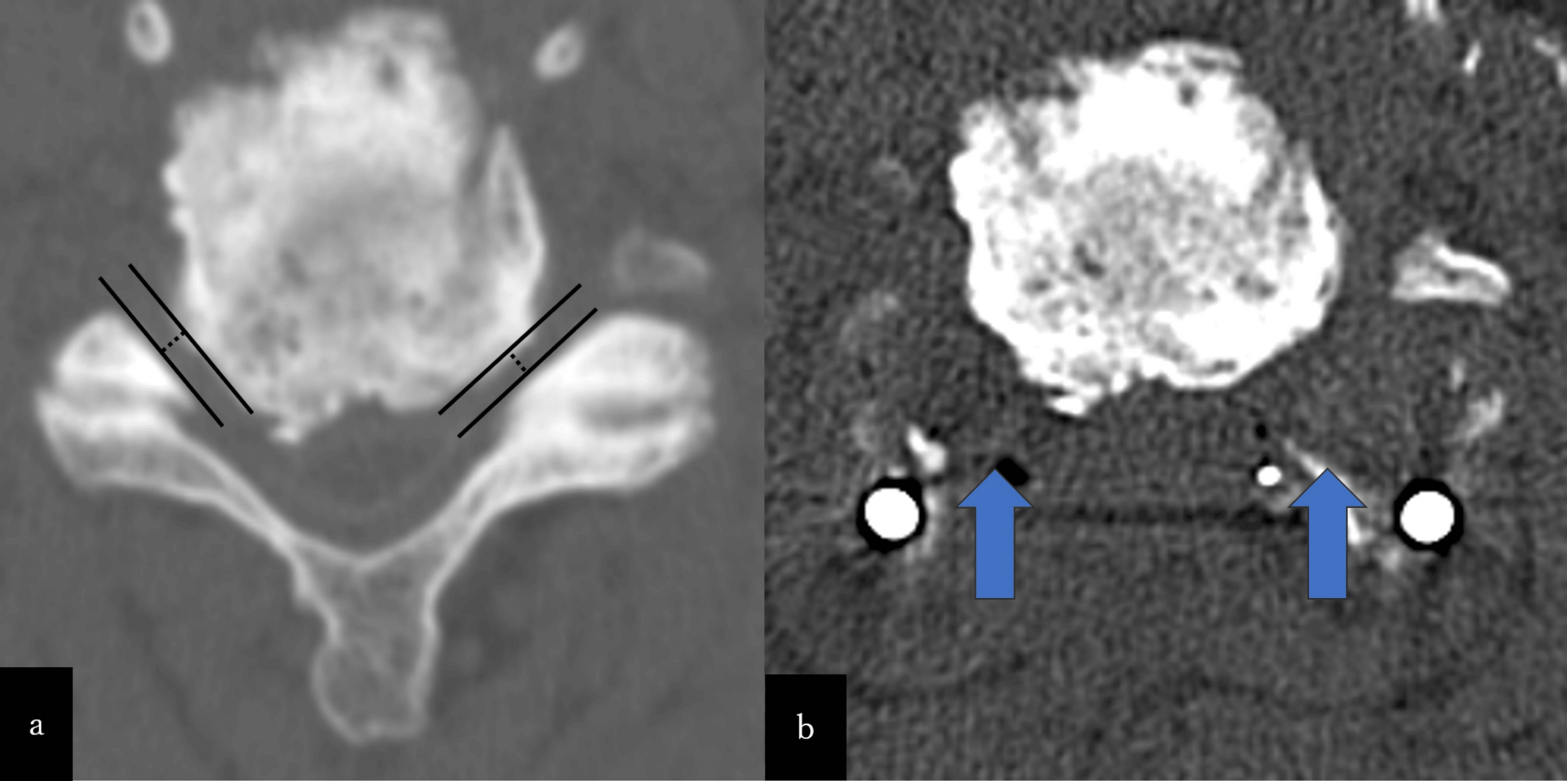
Axial CT at C4/5 (a) Preoperative image showing bilateral foraminal diameters measured at the narrowest point. (b) Post-facetectomy image showing a widened foramen on the operated side after facet resection.

Radiological evaluations were performed using lateral radiographs, CT, and MRI. On lateral radiographs, C2-7 cervical lordosis (CL) and segmental lordosis (SL) at C4/5 were measured preoperatively and postoperatively, and the change in each parameter (ΔCL and ΔC4/5 SL) was calculated. CL was defined as the angle between the inferior endplates of C2 and C7, and SL at C4/5 was defined as the angle between the superior endplate of C4 and the inferior endplate of C5. C4/5 foraminal diameter (FD) was measured on CT images using a 3D image analysis workstation (Ziostation; Ziosoft, Tokyo, Japan). Multiplanar reconstruction was used to obtain axial images parallel to the C4/5 endplate, and FD was measured at the narrowest portion of the C4/5 foramen (Figure 2). When bilateral foramina were evaluated, the smaller value was used for analysis. 

On MRI, the posterior shift of the spinal cord at C4/5 was assessed in accordance with previously described methods. Posterior shift of the spinal cord at C4/5 was quantified on midsagittal T2-weighted images as described by Mizutani et al. [17]. Intramedullary T2HI was evaluated at C3/4 and C4/5 on sagittal and axial T2-weighted MRI. T2HI was defined as visually identifiable intramedullary hyperintensity at the corresponding disc level. All radiological measurements and MRI assessments were performed by a single investigator.

Definition of C5 palsy and outcome classification

C5 palsy was defined as a postoperative decrease in deltoid muscle strength from a preoperative MMT grade of ≥3 to an MMT grade of ≤2 [18]. At the final follow-up, patients with C5 palsy were classified as having recovered C5 palsy, defined as an improvement in deltoid MMT to ≥3, or as having persistent C5 palsy, defined as a deltoid MMT remaining ≤2.

Subgroup analysis

A prespecified subgroup analysis was performed in patients with severe preoperative foraminal stenosis, defined as a C4/5 FD<2.6 mm. This cutoff was selected based on a previous systematic review and multivariate analysis that suggested future studies should evaluate patients with preoperative foraminal stenosis using this threshold [8].

Statistical analysis

All statistical analyses were performed using EZR. Continuous variables are presented as the mean ± standard deviation, and categorical variables are presented as counts and percentages. Between-group comparisons of categorical variables were performed using Fisher’s exact test, and comparisons of continuous variables were performed using the unpaired t-test. Risk factors for the development of C5 palsy were analyzed using multivariable logistic regression. Variables included in the primary multivariable model were selected based on clinical relevance, previously reported radiological risk factors, and the limited number of postoperative C5 palsy events. Given the possible influence of pathology on postoperative C5 palsy, an additional sensitivity analysis, including pathology (OPLL vs. CSM), was performed. A p-value < 0.05 was considered to indicate statistical significance.

## Results

Patient characteristics by C5 palsy status

A total of 94 patients met the inclusion criteria, and postoperative C5 palsy occurred in 20 (21.3%) patients. The distribution of underlying pathology tended to differ between patients with and without C5 palsy (CSM vs. OPLL: 16/4 vs. 41/33, respectively; p=0.07; Table 1). There were no significant differences in age, sex, or number of fused levels (Table 1). Facetectomy at C4/5 was performed at a similar frequency in patients with and without C5 palsy (9/20 (45.0%) vs. 39/74 (52.7%), p=0.62).

**Table 1:** Demographic and radiographic data by C5 palsy status Data are presented as the mean ± SD or n (%). p-values were calculated using the unpaired t-test for continuous variables and Fisher’s exact test for categorical variables (two-sided). p<0.05 was considered statistically significant. CL, cervical lordosis; CSM, cervical spondylotic myelopathy; FD, foraminal diameter; OPLL, ossification of the posterior longitudinal ligament; SL, segmental lordosis; T2HI, T2 high-signal intensity.

Variable	With C5 palsy (n=20)	Without C5 palsy (n=74)	p-value
Age (years)	68.0 ± 9.9	68.6 ± 10.6	0.83
Sex, female:male	6:14	25:49	1.00
Pathology, CSM:OPLL	16:4	41:33	0.07
Fused levels (n)	5.4 ± 2.4	5.2 ± 2.2	0.85
Facetectomy, n (%)	9 (45%)	39 (52.7%)	0.62
Preop FD right (mm)	3.33 ± 1.12	3.07 ± 1.03	0.34
Preop FD left (mm)	2.95 ± 1.03	3.25 ± 1.05	0.25
Posterior cord shift (mm)	2.70 ± 1.68	2.02 ± 1.80	0.15
C3/4 T2HI, n (%)	12 (60%)	23 (31.1%)	0.04
C4/5 T2HI, n (%)	10 (50%)	37 (50%)	1.00
Preop SL (°)	-1.40 ± 6.53	-1.38 ± 8.27	0.99
Postop SL (°)	3.19 ± 6.22	1.25 ± 5.94	0.20
ΔSL (°)	4.59 ± 4.45	2.63 ± 6.64	0.22
Preop CL (°)	7.78 ± 18.11	5.88 ± 15.17	0.67
Postop CL (°)	5.80 ± 14.54	7.69 ± 10.34	0.51
ΔCL (°)	-1.98 ± 11.55	1.81 ± 13.64	0.26

Radiological findings and risk factors for C5 palsy

Preoperative FD at C4/5 did not differ significantly between groups on either side (right: 3.33 ± 1.12 vs. 3.07 ± 1.03 mm, p=0.34; left: 2.95 ± 1.03 vs. 3.25 ± 1.05 mm, p=0.25; Table 1). In 8/20 (40.0%) patients with C5 palsy, the palsy developed on the side with a narrower preoperative foramen.

The amount of posterior cord shift at C4/5 was similar between the groups (2.70 ± 1.68 vs. 2.02 ± 1.80 mm, p=0.15; Table 1). Preoperative and postoperative C2-7 CL, C4/5 SL, and their postoperative changes did not differ significantly between patients with and without C5 palsy (Table 1). Intramedullary T2HI at C3/4 was more frequent in the C5 palsy group (12/20 (60.0%) vs. 23/74 (31.1%), p=0.04), whereas the frequency of T2HI at C4/5 did not differ between groups (10/20 vs. 37/74, p=1.00).

According to multivariable logistic regression, including T2HI at C3/4, facetectomy, posterior cord shift, and narrower-side C4/5 FD, only T2HI at C3/4 was independently associated with C5 palsy (OR: 3.11, 95% CI: 1.05-9.74; p=0.04). Facetectomy (OR: 0.45, 95% CI: 0.14-1.36; p=0.16), posterior cord shift (OR: 1.24 per 1-mm increase, 95% CI: 0.90-1.77; p=0.20), and FD (OR: 1.02 per 1-mm increase, 95% CI: 0.56-1.80; p=0.95) were not significantly associated with C5 palsy (Table 2). In an additional sensitivity analysis, including pathology (OPLL vs. CSM), pathology was not significantly associated with postoperative C5 palsy, and the direction and magnitude of the associations for the main variables were not materially changed.

**Table 2:** Multivariable logistic regression for C5 palsy CI, confidence interval; FD, foraminal diameter; OR, odds ratio; T2HI, T2 high-signal intensity.

Variable	OR	95% CI	p-value
T2HI at C3/4	3.11	1.05-9.74	0.04
Facetectomy	0.45	0.14-1.36	0.16
Posterior cord shift (per 1 mm)	1.24	0.90-1.77	0.20
FD (per 1 mm, narrower side)	1.02	0.56-1.80	0.95

Subgroup analysis of patients with severe foraminal stenosis

In the prespecified subgroup with severe foraminal stenosis (C4/5 FD<2.6 mm), baseline clinical characteristics (age, sex, pathology, and number of fused levels) did not differ between patients with and without C5 palsy (Table 3). The frequency of facetectomy did not differ significantly between patients with and without C5 palsy (Table 3). Radiological parameters, including preoperative FD, T2HI at C3/4 and C4/5, and posterior cord shift, were also not significantly different between groups (Table 3). Preoperative and postoperative C2-7 CL, C4/5 SL, and their postoperative changes also did not differ significantly between groups (Table 3).

**Table 3:** Severe foraminal stenosis subgroup (FD<2.6 mm) Data are presented as the mean ± SD or n (%). p-values were calculated using the unpaired t-test for continuous variables and Fisher’s exact test for categorical variables (two-sided). p<0.05 was considered statistically significant. CL, cervical lordosis; CSM, cervical spondylotic myelopathy; FD, foraminal diameter; OPLL, ossification of the posterior longitudinal ligament; SL, segmental lordosis; T2HI, T2 high-signal intensity.

Variable	With C5 palsy (n=11)	Without C5 palsy (n=37)	p-value
Age (years)	71.1 ± 7.4	70.8 ± 8.9	0.91
Sex, female:male	3:8	10:27	1.00
Pathology, CSM:OPLL	8:3	22:15	0.50
Fused levels (n)	6.0 ± 2.7	5.7 ± 2.1	0.70
Facetectomy, n (%)	7 (63.6%)	20 (54.1%)	0.73
Preop FD right (mm)	2.62 ± 0.85	2.38 ± 0.68	0.34
Preop FD left (mm)	2.32 ± 0.81	2.61 ± 0.91	0.34
T2HI at C3/4, n (%)	8 (72.7%)	15 (40.5%)	0.09
T2HI at C4/5, n (%)	5 (45.5%)	17 (45.9%)	1.00
Preop SL (°)	-1.95 ± 8.10	-2.31 ± 9.36	0.91
Postop SL (°)	4.43 ± 8.08	1.23 ± 6.57	0.18
ΔSL (°)	6.39 ± 4.84	3.54 ± 7.98	0.27
Preop CL (°)	5.58 ± 12.83	1.99 ± 18.87	0.56
Postop CL (°)	6.18 ± 12.67	7.51 ± 10.01	0.72
ΔCL (°)	0.60 ± 12.45	5.51 ± 15.70	0.35
Posterior cord shift (mm)	3.13 ± 1.53	1.95 ± 1.84	0.08

Association between facetectomy and recovery from C5 palsy in the severe foraminal stenosis subgroup

 Among the 11 patients with C5 palsy in the severe stenosis subgroup, recovery at the final follow-up was observed more frequently in patients who underwent facetectomy than in those who did not (100% (7/7) vs. 25% (1/4), p=0.02; Table 4).

**Table 4:** Outcome of C5 palsy in the severe stenosis subgroup according to whether facetectomy was performed p-values were calculated using Fisher’s exact test for categorical variables (two-sided). The dash indicates not applicable.

Group	Recovery (n)	Persistent (n)	p-value
Facetectomy	7	0	0.02
No facetectomy	1	3	―

## Discussion

In this single-center cohort of patients who underwent PCDF, including C4/5, the incidence of postoperative C5 palsy was 21.3%, within the upper range of previously reported rates for posterior instrumentation and decompression (4%-30%) [5-10]. Contemporary comparative evidence indicates that compared with anterior decompression, posterior approaches carry a higher risk of C5 palsy in DCM and that laminoplasty tends to yield lower C5 palsy rates than PCDF [1-4]. The incidence of C5 palsy in our study therefore aligns with the upper spectrum of posterior fusion-focused series.

Facetectomy at C4/5 did not reduce the incidence of C5 palsy in either the overall cohort or the prespecified severe-stenosis subgroup, echoing the mixed literature on prophylactic foraminotomy, in which benefits appear context dependent [12-16]. In contrast, in the small prespecified subgroup of patients with severe preoperative foraminal stenosis who developed C5 palsy, facetectomy was associated with a greater probability of recovery at the final follow-up. This finding should be interpreted as exploratory and hypothesis-generating because it was based on a small number of C5 palsy cases and a non-randomized surgical decision-making process. Although causality cannot be inferred, a plausible explanation is that wider posterior root decompression may alleviate residual tethering or compression that persists after posterior cord drift and contributes to prolonged postoperative root dysfunction despite initial decompression. Anatomical work also suggests that extended decompression beyond a standard foraminotomy can release tethering elements along the course of the root [19].

The definition of severe foraminal stenosis used in this study also warrants comment. We defined severe stenosis as a C4/5 FD<2.6 mm based on a threshold proposed in a previous systematic review and multivariable analysis of risk factors for postoperative C5 palsy [8]. This threshold was used as a prespecified operational definition rather than as a universally established diagnostic criterion. Nevertheless, prior studies have consistently identified narrower C4/5 FD as a risk factor for postoperative C5 palsy, supporting the clinical relevance of marked foraminal narrowing in this setting.

Multivariable analysis revealed intramedullary T2HI at C3/4 as an independent risk factor for C5 palsy. Together with prior reports linking cord signal changes and greater posterior cord drift to palsy [5-7,11], these findings support a multifactorial pathophysiology in which spinal cord vulnerability, potentially mediated by mechanisms such as ischemia-reperfusion injury, and nerve-root factors act in combination rather than in isolation [9,17,20]. From a practical standpoint, these findings suggest that future prevention strategies may need to consider both cord-level and nerve-root factors: (1) avoiding excessive posterior cord shift or excessive lordotic correction and (2) ensuring adequate foraminal decompression in high-risk foramina, while recognizing that even generous root decompression may not fully mitigate cord-level susceptibility.

The potential influence of sagittal alignment correction should also be considered. Previous studies have suggested that excessive correction of cervical kyphosis and postoperative changes in cervical alignment may contribute to C5 palsy. In the present cohort, however, C4/5 SL, C2-7 CL, and their postoperative changes did not significantly differ between patients with and without C5 palsy. In addition, all procedures were performed using a posterior-only approach with screw-rod fixation; no patient underwent anterior release or combined anterior-posterior surgery. During positioning, excessive correction was not intended, and the cervical spine was maintained as close to a neutral position as possible. These findings suggest that the occurrence of C5 palsy and the association between facetectomy and recovery observed in this cohort were unlikely to be primarily explained by differences in sagittal alignment correction.

Limitations of this study include the retrospective single-center design, the limited sample size, particularly in subgroup analyses, and potential selection bias regarding which patients received facetectomy. The indication for C4/5 facetectomy was not standardized during the study period and could not be fully reconstructed from historical medical records. Although facetectomy was generally considered based on preoperative C5-region symptoms, radiographic foraminal stenosis, and intraoperative judgment, these factors were not recorded in a standardized manner. Therefore, selection bias and confounding by indication could not be excluded, particularly in the severe stenosis subgroup. These limitations may have influenced both the incidence and recovery analyses, and causal inferences regarding the preventive or recovery-related effect of facetectomy cannot be drawn. Imaging measurements and clinical thresholds, such as stenosis severity, vary across studies, which complicates cross-study comparisons. Intra- and interobserver reliability analyses were not performed for radiographic measurements, which may have introduced measurement bias. Although C2-7 CL and C4/5 SL were evaluated and did not significantly differ between groups, the influence of sagittal alignment correction cannot be completely excluded. Furthermore, recovery was defined as improvement to MMT grade ≥3 rather than complete recovery to MMT grade 5. This definition was selected because the primary focus of this study was recovery from clinically apparent severe C5 palsy, not complete normalization of deltoid strength. Because some patients had preoperative deltoid weakness, requiring recovery to MMT grade 5 may have conflated recovery from postoperative C5 palsy with improvement of pre-existing neurological deficits. However, this definition may underestimate residual weakness in patients who improved from severe palsy but did not regain full strength. In addition, patient-reported functional outcomes were not collected; therefore, the impact of C5 palsy and its recovery on patient-perceived function and quality of life could not be evaluated. Prospective, adequately powered studies -- ideally with standardized indications for facetectomy, imaging definitions of foraminal stenosis and cord signal changes, and reliability assessments -- are warranted to clarify which patients, if any, derive preventive or recovery-related benefits from facetectomy and to further evaluate the exploratory association with improved recovery from C5 palsy.

## Conclusions

In this single-center retrospective cohort of patients who underwent PCDF including C4/5, C4/5 facetectomy was not associated with a lower occurrence of postoperative C5 palsy. However, in the small severe preoperative foraminal stenosis subgroup, facetectomy was associated with improved recovery after C5 palsy occurred. Intramedullary T2HI at C3/4 was independently associated with the development of C5 palsy. These findings do not support routine facetectomy for prophylaxis. The observed association between facetectomy and improved recovery in selected patients with marked foraminal stenosis should be interpreted as exploratory and hypothesis-generating, given the retrospective design, non-standardized indication for facetectomy, and small subgroup size. Prospective studies are needed to confirm these findings.
